# Modeling host-associating microbes under selection

**DOI:** 10.1038/s41396-021-01039-0

**Published:** 2021-06-22

**Authors:** Florence Bansept, Nancy Obeng, Hinrich Schulenburg, Arne Traulsen

**Affiliations:** 1 Max-Planck-Institute for Evolutionary Biology, Ploen, Germany; 2 Department of Evolutionary Ecology and Genetics, University of Kiel, Kiel, Germany

**Keywords:** Microbial ecology, Symbiosis, Theoretical ecology, Evolution, Microbiome

## Abstract

The concept of fitness is often reduced to a single component, such as the replication rate in a given habitat. For species with multi-step life cycles, this can be an unjustified oversimplification, as every step of the life cycle can contribute to the overall reproductive success in a specific way. In particular, this applies to microbes that spend part of their life cycles associated to a host. In this case, there is a selection pressure not only on the replication rates, but also on the phenotypic traits associated to migrating from the external environment to the host and vice-versa (i.e., the migration rates). Here, we investigate a simple model of a microbial lineage living, replicating, migrating and competing in and between two compartments: a host and an environment. We perform a sensitivity analysis on the overall growth rate to determine the selection gradient experienced by the microbial lineage. We focus on the direction of selection at each point of the phenotypic space, defining an optimal way for the microbial lineage to increase its fitness. We show that microbes can adapt to the two-compartment life cycle through either changes in replication or migration rates, depending on the initial values of the traits, the initial distribution across the two compartments, the intensity of competition, and the time scales involved in the life cycle versus the time scale of adaptation (which determines the adequate probing time to measure fitness). Overall, our model provides a conceptual framework to study the selection on microbes experiencing a host-associated life cycle.

## Introduction

Fitness is a central concept in evolutionary biology, of particular importance for the theory of natural selection. Fitness measures how well a phenotype performs in terms of reproductive success, i.e., in terms of its ability to survive and reproduce. Natural selection, acting through reproduction and inheritance of the phenotypic traits, then leads to an increase in the population of the genotypes producing high fitness phenotypes [1].

In any system, fitness emerges mechanistically from birth and death events [2]. However, when it comes to the study of particular experimental systems or models, the question of how to measure fitness is often delicate, and fitness is often defined from the outset, as a phenomenological parameter. For practical reasons, fitness is often quantified under controlled laboratory conditions, using different proxies such as a net replication rate measured over a limited period of time, or a proportion of habitats successfully colonized. But none of these fitness components alone provides a holistic view of what fitness encompasses in natural conditions. Indeed, in nature, individual lineages within a species are often subject to multi-step life cycles, during which they transition across different habitats (e.g., hosts and environments), which may each favor distinct life-history characteristics. Some of the steps of these life cycles allow for offspring production, others may be developmental, or may concern migration or dispersal to the appropriate environments, or mate finding – in the case of sexual reproduction (see for example [3] for multi-step life cycles in animals). Fitness of an individual lineage is thus a multivariate function of all the life-history traits characterizing its life cycle, and in particular, its reproduction rates within the habitats and, importantly, transmission across habitats.

The development of methods to take into account life cycles in the assessment of fitness has proven important in a variety of contexts. Historically, age-structured models have been developed to study human demography [4]. In the context of species conservation, or, at the other end of the spectrum, pest management, the focus has been on finding the “Achilles heels” of species life cycles to design efficient strategies to act upon them, in order to shape and preserve biodiversity [4]. This idea has further been developed theoretically, within the conceptual framework of metapopulation dynamics [5, 6]. Moreover, life cycle characteristics are also central to the study of the onset of multicellularity, to understand why and how group replication can be selected for [7, 8].

The question of how life cycle components contribute to fitness is of particular relevance for the study of microbial communities that associate with hosts (i.e., host-associated microbiota). Intricate life cycles are common in nature, where microbes can for example use hosts as vectors between different habitats [9, 10]. Having a living host as a habitat adds complexity to the assessment of fitness, given that the presence of the microbes may impact the host fitness and vice-versa. Research has often been biased towards the host perspective, and has focused on how microbes can contribute to host fitness by extending the host functional repertoire, e.g., performing digestive or immune tasks [11–13]. An exception is epidemiology and parasitology, that have specifically addressed the impact of the host fitness on the pathogen, in the form of trade-offs between transmission and within-host virulence [14–17]. But what about commensal relationships, where bacteria do not have a negative impact on the host fitness? In this context, what are the factors that determine fitness of a microbial lineage?

Here, we focus on a primary aspect of the impact of a host on the overall fitness of a microbial lineage, in that it provides the microbe with an alternative habitat, where growth conditions are potentially different from an environmental habitat. We propose a framework to assess the selection gradient acting upon the life-history traits of microbes undergoing a biphasic life cycle, in which they alternate between phases of host association and free-living environmental phases. Biphasic life cycles are likely to be at the origin of host-microbiota associations and are still widespread in current associations [18, 19]. We propose that the overall fitness for a microbial lineage during such a biphasic life cycle needs to integrate evolutionary success across the different steps of the life cycle. It is therefore shaped by reproductive rates in both of the habitats and additionally by the migration rates between the habitats. The gradient of selection determines the direction in the phenotypic space that evolution is expected to follow to maximize overall fitness. Our general aim is to provide a tool to compare the relative importance of the different life-history traits of a microbial lineage, starting only from the equations that describe the population dynamics experienced throughout the life cycle. We explore a simple continuous-time two-compartment model that allows microbes to migrate between a host and an environment. We use the method of sensitivity analysis [4] to infer how strongly the overall growth rate depends on the traits we are considering. In the baseline version of the model, we consider unconstrained growth. Subsequently, we extend our framework to include population size constraints. We define the local direction of the selection gradient as the optimal strategy for a microbial lineage to adapt to its life cycle, starting from the local values of the traits. We show the existence of defined regions of different optimal strategies in the phenotypic space in which it is either more beneficial to optimize growth or migration. The boundaries of these regions are driven by modeling assumptions such as competition, and the probing time chosen to measure fitness.

## Model

**Fig. 1:**
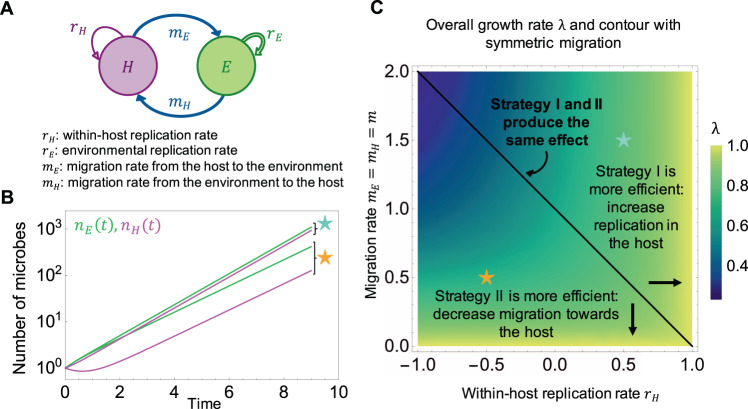
Optimal strategies in the baseline model (no competition). **A** Schematic diagram and definition of the rates for a microbial lineage migrating between a host and an environment and replicating in each compartment. For *r*_*E*_ > 0, the total number of microbes increases exponentially and we ask how the exponential growth rate can be increased by changing the parameters of the model. **B** Temporal dynamics of the number of microbes in each compartment, *n*_*E*_(*t*) and *n*_*H*_(*t*), for two different sets of traits values, indicated by colored stars in the traits space of panel **C**. The overall growth rate *λ* is the long-term slope of the curves. While the temporal dynamics contain the information on *λ*, they do not allow to distinguish between the different strategies on their own. **C** Overall growth rate *λ* (color scale) on the trait space determined by *r*_*E*_ = 1 (scaling time), *r*_*H*_ (x-axis) and *m* = *m*_*H*_ = *m*_*E*_ (y-axis). The overall growth rate *λ* is maximized for small *m* or for large *r*_*H*_. In addition, we focus on sensitivities, which capture how strongly the overall growth rate depends on the traits. The contour line shows the line of the traits space that equalizes the absolute values of the sensitivities derived analytically from equations [4] and [5], delimiting the regions of optimality of the two observed optimal strategies. When $\left| {\frac{{s_{m_H}}}{{s_{r_H}}}} \right| {\,}< {\,}1$, the optimal strategy is to increase the within-host replication rate *r*_*H*_ (strategy I). When $\left| {\frac{{s_{m_H}}}{{s_{r_H}}}} \right| {\,}> {\,}1$, the optimal strategy is to decrease the migration rate towards the host *m*_*H*_ (strategy II). The sensitivity to the third considered trait, $\left| {s_{m_E}} \right|$, is never larger than the two others on the considered traits space.

We focus on a single commensal microbial type and ask how the overall growth rate across its life cycle is affected by its life-history traits. We consider a simple biphasic life cycle, with two compartments corresponding to communicating habitats: a host and an environment. Let us write *n*_*H*_(*t*) for the number of host-associated microbes at a given time, and *n*_*E*_(*t*) for the number of environmental ones. We define the life-history traits of the microbial lineage as the rates at which individual microbes reproduce and die in each compartment, compete, and migrate from one compartment to another (Fig. 1A). The microbes reproduce clonally, and the net replication rates in the environment and within the host are *r*_*E*_ and *r*_*H*_, respectively. They could encompass both offspring production and death, and thus could be negative. The migration rates from the host to the environment and from the environment to the host are *m*_*E*_ and *m*_*H*_, respectively. We start with exponential growth. We later introduce intra-specific competition for space of intensity *k*_*ij*_ experienced by the microbes of compartment *i* due to the abundance of microbes in the compartment *j*. We assume that the number of microbes is large enough to be described by differential equations and assume that all rates introduced above are constant.

1 $$\left\{ {\begin{array}{*{20}{c}} {\frac{{\partial n_H}}{{\partial t}} = r_Hn_H + m_Hn_E - m_En_H - k_{HE}n_Hn_E - k_{HH}n_H^2} \\ {\frac{{\partial n_E}}{{\partial t}} = r_En_E + m_En_H - m_Hn_E - k_{EH}n_En_H - k_{EE}n_{E.}^2} \end{array}} \right.$$

In the following, we first consider unconstrained growth, where there is no competition (*k*_*EE*_ = *k*_*HH*_ = *k*_*EH*_ = *k*_*HE*_ = 0), before adding global competition (*k*_*EE*_ = *k*_*HH*_ = *k*_*EH*_ = *k*_*HE*_ = *k*), competition limited to one of the compartments (*k*_*EH*_ = *k*_*HE*_ = 0 and *k*_*EE*_ ≠ 0 or *k*_*HH*_ ≠ 0), and finally, equal competition in each of the compartments (*k*_*EH*_ = *k*_*HE*_ = 0 and *k*_*EE*_ = *k*_*HH*_ = *k*). While in nature it is likely that none of the *k*_*ij*_ vanishes and that a wide range of values are possible, the study of these limit cases gives powerful insights into what is to be expected in a wide range of situations.

## Results

### Baseline model: no competition

We start by assuming no competition and consider unconstrained growth in each of the two compartments. In this case, the equations describing our model become linear and can be rewritten in matrix form [4] as

2 $$\left( {\begin{array}{*{20}{c}} {\frac{{\partial n_{H}}}{{\partial t}}} \\ {\frac{{\partial n_{E}}}{{\partial t}}} \end{array}} \right) = \underbrace{\left( {\begin{array}{*{20}{c}} {r_{H} - m_{E}} & {m_{H}} \\ {m_{E}} & {r_{E} - m_{H}} \end{array}} \right)}_{{\mathrm{projection}}\, {\mathrm{matrix}}}\left( {\begin{array}{*{20}{c}} {n_{H}} \\ {n_{E}}\end{array}} \right)$$

The dominant eigenvalue *λ* of the above-defined projection matrix gives the asymptotic overall growth rate of the considered microbial lineage. This quantity is an appropriate measure of fitness [4] insofar as it measures reproductive as well as transmission success and recapitulates the effects of all the life-history traits (*r*_*E*_, *r*_*H*_, *m*_*E*_, and *m*_*H*_, also defining the phenotype in our model). Overall microbial fitness is thus integrated across the different steps of the life cycle, thereby considering the reproductive rates (i.e., replication rates) within each of the compartments and importantly transmission rates (i.e., migration rates) across the compartments. The dominant right eigenvector represents the stable distribution of microbes in the two compartments, and the number of microbes in each of the compartments grows exponentially with rate *λ*. The value of *λ* can be calculated at each point of the phenotypic space defined by the ranges of possible values that could be taken by the life-history traits *r*_*E*_, *r*_*H*_, *m*_*E*_, and *m*_*H*_. The dependence of *λ* on these traits tells us at which points of the phenotypic space fitness is maximized and how it can be increased at all other points.

From the projection matrix, we calculate the dominant eigenvalue as

3 $$\lambda = \frac{1}{2}\left(\sqrt {\left( {r_E + r_H - m_E - m_H} \right)^2 {\,}-{\,} 4\left( {r_Er_H - r_Em_E - r_Hm_H} \right)} + r_E +r_H - m_E - m_H \right).$$

Note that if microbes replicate at the same rate in the host and in the environment, i.e., if *r*_*E*_ = *r*_*H*_ = *r*, *λ* simplifies to *r*, regardless of the migration rates *m*_*H*_ and *m*_*E*_. When there is an asymmetry between the two replication rates however, which is very likely to be the case in nature, then the migration rates also affect the overall growth rate. In the following sections, we study this effect compared to the effect of the replication rates. We arbitrarily set *r*_*H*_ ≤ *r*_*E*_, and *r*_*E*_ > 0 – otherwise the lineage goes extinct. In biological terms, this corresponds to the situation where the microbial lineage is initially more adapted to the environment than to the host and thus grows faster in the environment. But mathematically, in this model, host and environment are symmetrical, i.e., they only differ by the rates defined above. Thus, the chosen direction of this inequality does not carry any strong meaning, and there is no loss of generality in making this choice. In particular, one can access the opposite biological situation where microbes replicate faster in the host than in the environment – as is the case for viruses, that can only replicate in the host (*r*_*H*_ > 0) but decay in the environment (*r*_*E*_ < 0) – by a single switch of the index *E* and *H*.

Let us first study the case where the migration rates from and towards the environment are equal, i.e., *m*_*E*_ = *m*_*H*_ = *m* > 0. Setting *r*_*E*_ = 1 to scale time (and thus, measuring all other rates in units of the replication rate of the microbe in the environment), *λ* reduces to

4 $${\uplambda}_{sym} = \frac{1}{2}\left( {1 + r_H - 2m + \sqrt {\left( {1 - r_H} \right)^2 {\,}+ {\,}4m^2} } \right)$$

For any fixed positive value of *m*, *λ*_*sym*_ is a strictly increasing function of *r*_*H*_, which reflects the fact that increasing *r*_*H*_ allows for additional growth within the host. We will limit ourselves to the study of *r*_*H*_ ≥ −1, which ensures a positive value for *λ*_*sym*_. For any fixed value of *r*_*H*_, *λ*_*sym*_ is a decreasing function of *m*, which reflects the fact that for increasing *m*, microbes are increasingly lost towards the host, where growth is slower than in the environment. Figure 1C shows the value of *λ*_*sym*_ on the reduced phenotypic space defined by *r*_*H*_ and *m*. The maximum possible value for *λ* is 1 (in units of *r*_*E*_). This value is achieved either by increasing the ratio of replication rates between host and environment, so that the replication rates in both compartments are identical (strategy I), or by reducing migration between host and environment, and in particular, by reducing *m*_*H*_ (strategy II). This second strategy allows microbes to spend a longer time in the environment on average. Note however, that this strategy is limited, since setting *m* to zero decouples the two compartments completely, in which case the microbial lineage is no longer subject to a multi-step life cycle.

How strong is the selection on these traits? This question can be approached by inferring how strongly the overall growth rate depends on the traits we are considering. One standard approach to measure this is sensitivity analysis [4]. One defines the sensitivity of the overall growth rate *λ* achieved by the phenotype described by the vector x = (*x*_1_,…, *x*_*N*_) in the trait space to its *i*th life-history trait as

5 $$s_{\mathrm{i}}\left( {\mathbf{x}} \right) = \left. {\frac{{\partial {\uplambda}}}{{\partial {\mathrm{x}}_{\mathrm{i}}}}} \right|_{\mathbf{x}}$$

This quantity gives the change in the value of *λ* that results from a small increment of the trait *i*. It is a local property that can be calculated for each point ${\mathbf{x}}$ of the trait space. The vector of the sensitivities at point ${\mathbf{x}}$ gives the direction of the selection gradient on the fitness landscape. In other words, to achieve efficient phenotypic adaptation, the lineage should move in the trait space following the direction of this gradient.

If the lineage can invest in phenotypic adaptation only by tuning one of its life-history traits at a time, then it should act upon the trait that has the largest (absolute) sensitivity at the current position of the lineage in the trait space. In our model, in all generic cases (i.e., when *m* > 0), the largest sensitivity is always associated to the increase of the trait *r*_*E*_, the replication rate in the fast-growing compartment. However, we assume that the considered microbial lineage is initially fully adapted to the environment, so that it has reached its evolutionary limit, and we can essentially ignore the sensitivity to *r*_*E*_ throughout the manuscript to focus on the sensitivity to the other traits. This reasoning allows to divide the trait space into regions of distinct optimal strategies, as shown in Fig. 1C. In the regime of high migration rates (i.e., when the switch between the compartments is so rapid that the microbial lineage is almost experiencing a habitat having average properties between the host and the environment), strategy I (increasing *r*_*H*_) becomes almost always optimal, except for small replication ratios, where there is almost no replication in the host. In summary, migration rates are important when replication in the host is slow compared to the environment, and when migration itself is slow. These conclusions remain qualitatively unchanged with asymmetric migration rates, although a third optimal strategy (increasing *m*_*E*_) appears for an intermediate region of the traits space when the asymmetry is important (see electronic Supplementary Material (ESM) section 1 and Supplementary Fig. S1).

### Model with global competition between all microbes

In the baseline model, there are no constraints on growth. In nature, however, microbes do face limits to their growth. Since the equations above are linear and can only give rise to exponential growth or exponential decay, they can only describe the microbial dynamics over a limited period of time. In order to account for saturation and competition during growth, we thus need to introduce non-linear terms to the equations (1). The study of this kind of systems often focus on long-term dynamics, yet it can be of high practical relevance to study the transient optimal strategies, as shorter timescales are often relevant in the real world – whether it be due to experimental constraints or to ecological disturbances and perturbations [20]. Since we are going to consider some out-of equilibrium dynamics, in particular in the section with competition limited to one of the compartments, and because we are also interested in transient properties, we will adopt a numerical approach based on the number of microbes [21, 22].

In this section, we study the case of a microbial lineage constrained by global competition occurring at rate *k* = *k*_*HH*_ = *k*_*EE*_ = *k*_*EH*_ = *k*_*HE*_. This situation could correspond to a host-associated microbe living in direct contact with an external environment, e.g., on the surface of an organism. Alternatively, what we call the “environment” in our model could represent another host compartment in direct contact with the other, like the gut lumen and the colonic crypts. In that case, microbes living in association with the host are in direct contact with those in the environment and can mutually impact each other’s growth. This is of particular relevance if microbes living in both compartments rely on and are limited by the same nutrients for growth.

**Fig. 2:**
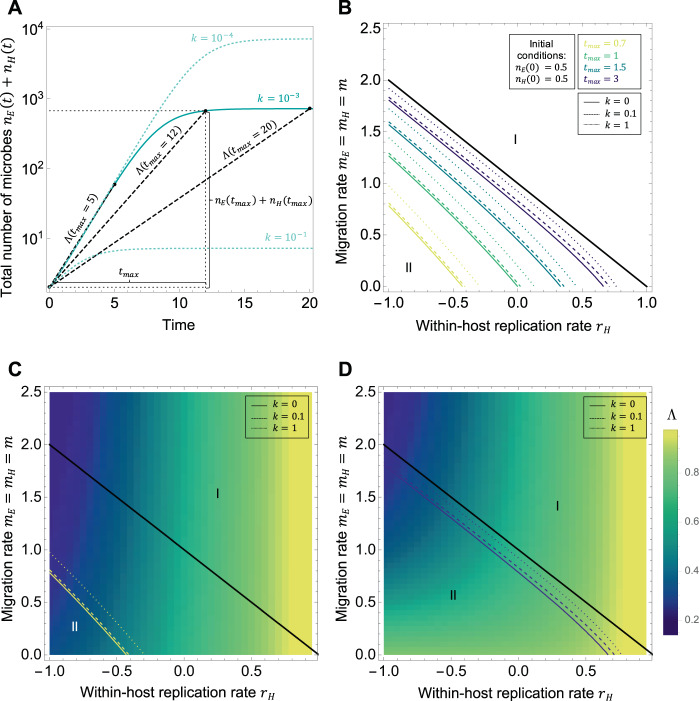
Optimal strategies in the model with global competition. **A** Temporal dynamics of the total number of microbes *n*_*E*_(*t*) + *n*_*H*_(*t*) for three different sets of traits values, differing only by their intensity of competition *k* = *k*_*HH*_ = *k*_*EE*_ = *k*_*EH*_ = *k*_*HE*_. Other parameter values are: *r*_*H*_ = 0.1, *m*_*E*_ = *m*_*H*_ = 0.5. The effective overall growth rate *Λ* is calculated numerically by taking the slope of the straight line that connects the abundances in *t* = 0 and in *t*_*max*_, thus making *Λ* a quantity that strongly depends on *t*_*max*_. **B** Change in the contour line delimiting the regions of optimality of the two optimal strategies (strategy I: increasing *r*_*H*_; strategy II: decreasing *m*_*H*_) with *t*_*max*_, the time chosen to measure the final number of microbes, measured in units of 1/*r*_*E*_. Initially the microbes are equally distributed between the host and the environment. Supplementary Fig. S2 shows how this is modified with different initial conditions. Because in this model all the microbes are equally impacted by competition, with *t*_*max*_ large enough, one recovers the contour line of the baseline model calculated analytically (black line). Continuous lines: *k* = 0, i.e., no competition. Dashed lines: increasing values of *k* (competition intensity). **C**, **D** Change in the fitness landscape with *t*_*max*_ (panel **C**: *t*_*max*_ = 0.7 and panel **D**: *t*_*max*_ = 3). The colored lines show the contour delimiting the regions of optimality of strategies I and II for three different values of *k*, as shown on panel B. Black line: long-term limit of no competition from the base model.

From the microbial abundances in the two compartments obtained by numerically solving the equations, one can build a proxy for the overall growth rate of the microbial lineage. To remain consistent with the previous section, we define

6 $$\varLambda \left( {\mathbf{x}} \right) = \frac{1}{{t_{max}}}\log \left( {\frac{{n_E\left( {t_{max}} \right) + n_H\left( {t_{max}} \right)}}{{n_E\left( 0 \right) + n_H\left( 0 \right)}}} \right)$$

i.e., the effective exponential growth rate of the microbial lineage over a chosen period of time [0, *t*_*max*_]. Figure 2A provides a graphical explanation for the expression of *Λ*. There are indeed several fundamental differences between the effective exponential growth rate *Λ* in a non-linear system and the asymptotic growth rate *λ* in a linear system, the dominant eigenvalue of the projection matrix as defined in the baseline model. First, *Λ* provides a measure of growth for the whole lineage, but is not an asymptotic growth rate (as compared to *λ* in the baseline model): in the case of global saturation, replication stops when the carrying capacity is reached, and the asymptotic growth rate for the whole lineage would thus be zero. Therefore, the choice of the probing time *t*_*max*_ has an impact on *Λ*, as shown in Fig. 2A. Second, the choice of the exact form of *Λ* now implies biological assumptions on the selection pressure experienced by the microbial lineage: choosing the effective exponential growth rate over the whole lineage as we do implies that selection is acting on both compartments evenly. There may be some situations in which the microbes in one of the compartments only are artificially selected for (e.g., as part of the protocol of an evolution experiment). In such cases, it would make sense to define *Λ* as the effective exponential growth rate over just this compartment. This may lead to different conclusions, in particular at the transient scale. One must thus adapt *Λ* to the specifics of the modeled system. In addition, the choice of *t*_*max*_ itself has a biological meaning, and should in particular not exceed the time upon which the dynamics of the system are accurately described by the set of equations. This may also be determined by experimental times.

We now calculate the sensitivity of *Λ* in the direction of the trait *i* at the point x of the phenotypic space as

7 $$S_i = \frac{{\varLambda \left( {x_1,x_2, \ldots ,x_{i - 1},x_i + \delta x_i,x_{i + 1}, \ldots ,x_N} \right) - \varLambda \left( {x_1,x_2, \ldots ,x_N} \right)}}{{\delta x_i}}$$

with *δx*_*i*_ the discretization interval, and *N* the number of traits defining a phenotype x.

For this numerical approach, additional choices need to be made. First, the trait space needs to be discretized. Then, to calculate Eq. (7), one needs to choose a set of initial conditions and a probing time at which to measure the microbial abundances, as exposed in detail for the linear case in [20]. Finally, we need to choose the discretization interval *δx*_*i*_. In the following, we always choose *δx*_*i*_ sufficiently small for convergence, i.e., so that it does not significantly impact the numerical values of the sensitivities, and focus on the choices of the other parameters (probing time and initial conditions) and the influence of the competition intensity *k*. One strategy to explore the possible impact of initial conditions is to use “stage biased vectors” [20], i.e., extreme initial distributions of microbes across the two compartments. This corresponds to initial conditions where microbes either exist only in the host or only in the environment.

In Fig. 2B, we show how the contour lines delimiting the two optimal strategies change with the final time *t*_*max*_ chosen to measure the overall growth rate and with the intensity of competition *k*, for a mixed initial condition (*n*_*E*_(0) = 0.5, *n*_*H*_(0) = 0.5), and Supplementary Fig. S2 shows how this is modified with stage biased vectors. In all cases, with sufficiently long *t*_*max*_, the contours converge to the contour plot of the baseline model shown in the previous section. This is expected, since competition here affects all the microbes in the same way, so that the equilibrium distribution is the same as the asymptotic distribution of the baseline model (given by the dominant eigenvector). Mathematically, global competition can be seen as a modification of the baseline projection matrix by subtracting an identity matrix times a scalar depending on time. This does neither affect the eigenvectors nor the dependence of the dominant eigenvalue on the traits.

In the case where all the microbes are initially in the environment (Supplementary Fig. S2A), there is no transient effect and whichever *t*_*max*_ is chosen, all the contour lines collapse to the limit of the baseline case. In the case where all the microbes are initially in the host (Supplementary Fig. S2B), a third optimal strategy transiently appears (increasing *m*_*E*_) and remains at long times around *m* = 0. In this unfavorable condition (*m* = 0 and an initially empty environment), increasing the microbial flux towards the environment becomes more important than limiting the flux of microbes leaving it (which is nonexistent when *m* = 0).

Finally, we observe that the intensity of competition has only a small effect on the contours (Fig. 2B and S2B), but increasing *k* appears to slightly accelerate convergence to the baseline contour. By limiting growth in the host compartment – when it is initially relatively more populated than in the asymptotic distribution – competition facilitates the convergence to the baseline asymptotic distribution, where most of the microbes live in the environment.

### Model with competition within one of the compartments only

In this section we consider competition happening inside one of the compartments only (i.e., *k*_*EH*_ = *k*_*HE*_ = 0 and *k*_*EE*_ ≠ 0 or *k*_*HH*_ ≠ 0). We will start by considering competition in the host only (the slow-replicating compartment). In a second step we also look at the case with competition limited to the environment. One should bear in mind that it also covers the case of competition limited to a host where replication is faster than in the environment (*r*_*H*_ > *r*_*E*_), provided a switch of the *H* and *E* index.

In the case where competition is limited to only one of the compartments, we do not expect an equilibrium to exist for all traits combination of the phenotypic space. If migration is not sufficiently important, the number of microbes in the unconstrained compartment keeps increasing exponentially faster than the number of microbes in the constrained compartment, which contribution to the whole lineage thus becomes rapidly negligible. At sufficiently high migration rates however, an equilibrium is expected, because microbes switch habitats sufficiently rapidly for competition to be globally effective, although it directly affects only one of the compartments.

#### Competition in the host only (slow-replicating compartment)

**Fig. 3:**
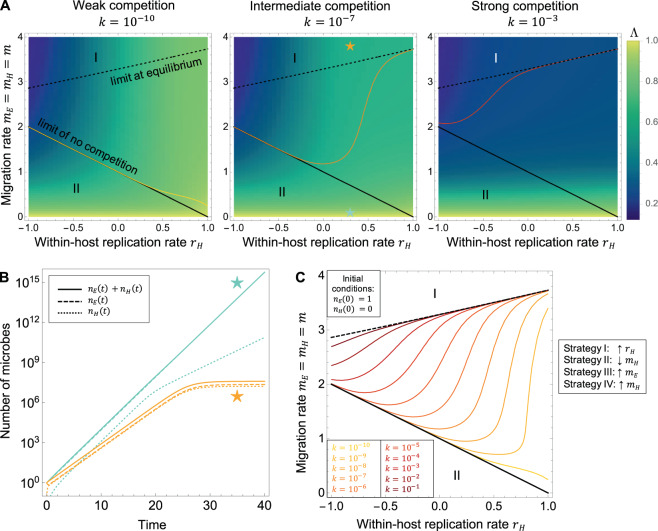
Optimal strategies in the model with competition in the host only. **A** Change in the fitness landscape with the within-host competition intensity *k* = *k*_*HH*_. Colored lines: contours of equal sensitivities delimiting the optimal strategies (as shown in panel **C**). Black lines: limit contours (solid line: limit of no competition, from the baseline model; dashed line: limit derived from the number of microbes at equilibrium). Other parameters: *t*_*max*_ = 30, *n*_*E*_(0) = 1, *n*_*H*_(0) = 0. **B** Number of microbes in function of time, for the parameter combinations indicated by colored stars in panel **A**. When there is competition in the host only, there is an equilibrium only if migration is important enough. **C** Change in the contour lines delimiting the regions of optimality of the strategies with increasing *k* (within-host competition intensity). All the microbes are initially in the environment (*n*_*E*_(0) = 1, *n*_*H*_(0) = 0). Solid colored lines: limit between the regions of optimality of strategy I (increasing *r*_*H*_) and II (decreasing *m*_*H*_). Other parameter: *t*_*max*_ = 30. The region of optimality of strategy I tends to narrow down and shifts out of the *m* < 1 region to converge to the contour of equal sensitivities of the number of microbes at equilibrium (thin dashed line).

When there is competition in the host only, there is no (positive) equilibrium for all *m*_*H*_ < *r*_*E*_ = 1 (Fig. 3B). In this case, replication inside the host should have less importance for the lineage because the number of microbes associated to the host becomes negligible compared to the ones present in the environment. In this region of the phenotypic space we thus expect the sensitivity of *Λ* to the parameter *r*_*H*_ to tend to zero with increasing probing times *t*_*max*_ or intensity of competition *k* = *k*_*HH*_, whatever be the other parameters (initial conditions, intensity of competition). When migration out of the environment is sufficiently important for an equilibrium to exist, we can derive the expression of the number of microbes at equilibrium analytically and perform a sensitivity analysis to determine the limit of the contour line separating the regions of optimality of the different strategies.

Figure 3 verifies these verbal arguments. As expected, for a fixed *t*_*max*_, we recover the shape of the fitness landscape of the baseline model for small values of *k* = *k*_*HH*_. When increasing *k*, the values of *Λ* become smaller overall: growth is slower due to competition. For small *k* values, the contour delimiting strategy I from II is close to the baseline limit: the effect of competition is negligible. With increasing values of *k*, strategy I (increasing *r*_*H*_) sees its area of optimality reduced out of the *m*_*H*_ < *r*_*E*_ = 1 region, until the contour converges to the limit of equal sensitivities of the number of microbes at equilibrium (Fig. 3A, B, and Supplementary Fig. S3).

When initially the microbes are in the host only (Supplementary Fig. S3A, C), we can again observe the appearance of the third strategy (increasing *m*_*E*_), around *m* = 0. Indeed, when *m* = *m*_*E*_ = *m*_*H*_ = 0 initially, decreasing *m*_*H*_ (strategy II) has no effect, while increasing *m*_*E*_ will allow the colonization of the initially empty environment.

Finally, the impact of increasing the probing time *t*_*max*_ at fixed *k* is similar in every way to increasing the competition intensity *k* at fixed *t*_*max*_ (Supplementary Fig. S3B, C).

#### Competition in the environment only (fast-replicating compartment)

When there is competition in the environment only, there is no (positive) equilibrium for all *m*_*E*_ < *r*_*H*_. In this region of the phenotypic space, the number of microbes in the environment becomes substantially smaller than the number present in the host after some time. As a consequence, strategy I (increasing the replication rate within the host) becomes more important, so that we see its area of optimality extend, see Supplementary Fig. S4. For a fixed *t*_*max*_, with a small value of *k* we recover the shape of the fitness landscape from the baseline model with no competition, but increasing *k* shifts the contour line to lower *r*_*H*_ until the strategy II (decreasing *m*_*H*_) disappears from the *m*_*E*_ < *r*_*H*_ region and the delimitation of the strategies approaches the contour of equal sensitivities of the number of microbes at equilibrium, calculated analytically. Remarkably, we also observe the appearance of a fourth optimal strategy around *m* = 0, increasing *m*_*H*_. Intuitively, initial conditions where all the microbes are initially located in the (fast-replicating) environment are less favorable when there is competition in the environment, so that migration towards the host (where growth remains unconstrained) becomes more important when the migration rates are initially small. Similar to the previous case, when initially microbes are in the host only (Supplementary Fig. S5A, C), the third strategy (increasing *m*_*E*_) prevails around *m* = 0. As before, the impact of increasing the probing time *t*_*max*_ at fixed *k* is similar in every way to increasing the competition intensity *k* at fixed *t*_*max*_ (Supplementary Fig. S5B, C).

### Competition of equal intensity within each compartment

**Fig. 4:**
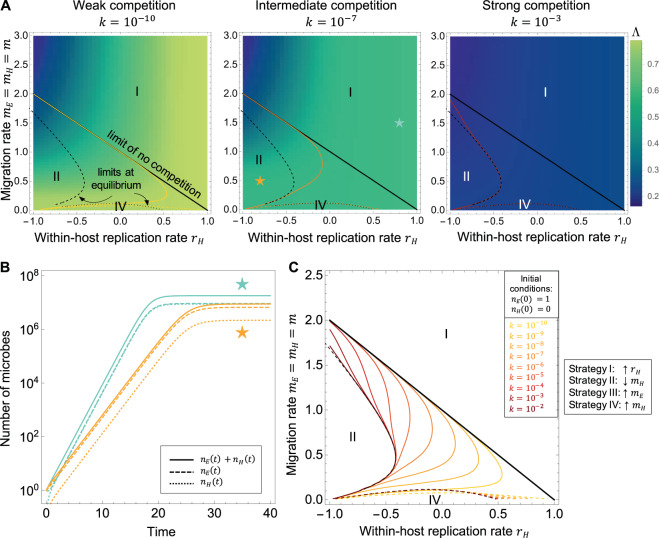
Optimal strategies in the model with equal intensity of competition within each compartment. **A** Change in the fitness landscape with the competition intensity *k* = *k*_*HH*_ = *k*_*EE*_. Colored lines: contours of equal sensitivities delimiting the optimal strategies (as shown in panel **C**): solid line, between strategies I and II; dashed line, between I and IV. Black lines: limit contours (solid line: limit of no competition, from the baseline model; dashed and dotted lines: limit derived from the number of microbes at equilibrium). Other parameters: *t*_*max*_ = 30, *n*_*E*_(0) = 1, *n*_*H*_(0) = 0. **B** Number of microbes in the two compartments in function of time, for the parameters combinations indicated by colored stars in panel **A**. When there is competition of same intensity in each of the compartments, there always exists a positive equilibrium. **C** Change in the contour lines delimiting the regions of optimality of the strategies with increasing *k* (competition intensity). All the microbes are initially in the environment (*n*_*E*_(0) = 1, *n*_*H*_(0) = 0). Solid colored lines: limit between the regions of optimality of strategy I (increasing *r*_*H*_) and II (decreasing *m*_*H*_); dashed colored lines: between strategy I and IV. Other parameter: *t*_*max*_ = 30. The region of optimality of strategy I tends to expand until it converges to the contour of equal sensitivities of the number of microbes at equilibrium (black dashed line). The black dotted line is also derived from the number of microbes at equilibrium and delimits the area of optimality of strategy IV.

When there is competition of equal intensity in the host and the environment (i.e., *k*_*EH*_ = *k*_*HE*_ = 0 and *k*_*EE*_ = *k*_*HH*_ = *k*), we observe very similar results to the previous section, with competition in the environment only (see Fig. 4 and Supplementary Fig. S6): increasing *k* or increasing *t*_*max*_ leads to the disappearance, at long times, of the area of optimality of strategy II (decreasing *m*_*H*_), except for a distinct region of small *r*_*H*_ and intermediate *m*, predicted by the contour of equal sensitivities of the number of microbes at equilibrium. Strategy IV (increasing *m*_*H*_) is optimal around *m* = 0. This implies that the effect of competition in the fast-replicating compartment has a dominating effect on the overall growth rate.

## Discussion

Out in the wild, microbial lineages are often subject to multi-step life cycles, where they alternate between at least two habitats. Each of the steps of these life cycles can contribute to the overall reproductive success. In general, microbial fitness is thus more complex than the common approximation of growth yield used in the lab. This is particularly true for microbes with life cycles that involve a host-associated phase and a free-living phase, as commonly observed for many host-associated microbiota members [19]. In this case, selection should favor traits which ensure both high reproductive rates within each habitat, but also successful transmission between them. A framework to study fitness in all its complexity is needed in the field of microbiota studies, which could benefit from some of the mathematical tools first introduced in demography, as the ones used in this work. Here, we investigate a model of a microbial lineage living, replicating, migrating, and competing in and between two compartments: a host – assumed to be, throughout the paper, a compartment where replication is slower – and an environment. To analyze the selection gradient experienced by the microbial lineage going through this biphasic life cycle – with phases in the environment and phases in the host – we perform sensitivity analysis. We focus on the leading direction of the selection gradient at each point of the phenotypic space, thereby defining an optimal strategy for the microbial lineage to maximize its fitness.

We show that in the case of unconstrained exponential growth in both the compartments, there are two optimal strategies: increasing the replication rate in the host compared to the environment (strategy I), and decreasing the migration rate to the host (strategy II) to maximize the time spent in the fast-replicating compartment. The first strategy is optimal at initially high within-host replication rates and high migration rates, while the second strategy is optimal at initially small migration rates and small within-host replication rates.

Next, we extend the model to a scenario where microbial growth is limited by competition. We start with global competition, a case which could describe competition for a resource homogeneously shared between the host and the environment. Biologically, this corresponds to communities of microbes that are associated with hosts, but have extensive contact with the environment, as the skin or other epithelial microbiota for example [23, 24]. In this case, we show that apart from a transient effect, the optimality of the strategies is conserved from the case without competition. With competition in the host only (the slow-replicating compartment), at longer probing times, or at higher competition intensities, the strategy I (increasing the ratio of replication rates) is disfavored when migration out of the environment is slower than replication in the environment, i.e., where there is no equilibrium. Strategy II (decreasing migration to the host) thus increases its area of optimality. Inversely, with competition in the environment only (the fast-replicating compartment), or with competition of equal intensity within the host and within the environment, the strategy II is disfavored when migration out of the host is slower than replication in the host, leaving strategy I as the only optimal strategy in this region of the parameter space. Unsurprisingly, this suggests that competition within the fast-replicating compartment dominates the effect on the selection gradient.

While this analysis provides crucial information on the selection gradient that shapes microbial adaptation to life cycles involving host association, it does not take into account the evolvability of the traits themselves. Although the selection gradient is a good indicator of the expected evolutionary path in the phenotypic space, the underlying genotype/phenotype mapping does not always allow for this path to be taken [25–28], and the outcome of evolution may thus be different. The discrete nature, the non-additivity and non-linearity of genetic information, as well as the existence of costs, trade-offs and evolutionary constraints may prevent the predicted continuous change on the phenotypic trait. In addition, using sensitivities is built on the assumption that adaptation generates additive changes in life-history traits. Although this is a common assumption, different choices are sometimes made. For example, multiplicative changes of the traits are assumed in elasticity analysis [4, 21, 27, 29], which presents the advantage of manipulating only proportional changes and thus non-dimensional quantities, but deals poorly with traits that can take the value of zero. These fundamental assumptions can sometimes result in different inferred selection gradients, as was shown for example in the context of age-classified populations [30].

Stepping back, we can evaluate the predictions of our model in the light of biological observations. Evolution experiments where microbial lineages are serially passaged through a host and an environment are of particular interest here, to assess the response to selection resulting from biphasic life cycles. The key role of microbial immigration during the initial adaptation to their zebrafish host has for example been highlighted in [31]. In *Drosophila* [32] and in *C. elegans* [33], experimental selection towards host association resulted in adaptive changes in microbial life history with a direct impact on host fitness. In detail, in the first case, there is evolution towards by-product mutualism, and in the second, which concerns an initially pathogenic population, evolution towards less virulence and an increased carrying capacity.

Conceptually, using an integrative, overall growth rate as a measure of fitness across the life cycle provides a complementary insight to invasion fitness approaches [34, 35] developed to analyze such evolution experiments, for example in [36, 37]. While invasion fitness analysis relies on assessing the long term chances of successful invasion of an established population at equilibrium by a new mutant strain of defined traits values, sensitivity analysis of the overall growth rate provides a systematic framework that can be applied to out-of-equilibrium systems, and provides information on shorter time scales. Both frameworks rely on different proxies to assess a fitness capturing its different components - in one case, the frequency of patches where the microbe is present, and in the other, the overall growth rate, but both frameworks converge on the key role of migration between compartments. In fact, in many common cases like global competition, the long-term predictions of invasion fitness are recovered with the sensitivity analysis of the effective growth rate by setting *t*_*max*_ sufficiently large [21].

In future work, our framework could be extended in different directions to capture additional characteristics of microbial life cycles in host association. The first extension could be to increase the number of compartments. While the question of fluctuating environments has been studied before, in discrete times or in a different context [8, 21], in our context it may be profitable to consider and include host population dynamics. This would notably allow us to include microbial traits that affect host fitness in our analysis. A second direction could be to include non-homogeneities and stochasticity. A first step could be to introduce several interacting taxa with different life-history traits, and assess how the presence of additional taxa potentially modifies the selection on the taxon of focus. Secondly, our deterministic description is valid only if the number of microbes is sufficiently large at all times and can only describe the average selection gradient experienced by the lineage. Introducing stochasticity would crucially allow the study of differentiation, which may play an essential role in the response to multi-step life cycles which include replication in several steps. Differentiation, in the form of speciation, phenotypic plasticity, or bet-hedging is indeed observed in evolution experiments and natural microbial populations [38–43]. It is also observed in host-associated populations [44] and may thus be expected in evolution experiments that include a host-association phase. In a stochastic setting with mutation of the life-history traits, it could be important to also incorporate other mechanisms of transfer of genetic information, such as horizontal gene transfer and recombination, which could decelerate or even prevent differentiation [45, 46]. Finally, a key aspect that we have so far excluded is spatiality. Effects of spatiality on the selection gradient are known for example in a simple Petri dish system, where the existence of an optimal expansion speed for a given habitat size is shown [47, 48]. Generally, hosts are highly structured habitats with variation in nutrients and chemical and physical gradients shaping for example the gut [49–51], which may also favor differentiation. The introduction of several compartments or sub-compartments within the hosts could represent a first step in this direction.

In conclusion, the framework we introduce here with a minimal model provides a basis to study the consequences of habitat switching for microbes, and will allow to explore additional aspects of host association in the future. It meets the need to conceptualize fitness as a holistic measure that captures all the aspects of microbial life cycles. With the development of this framework, we aim to contribute to a better understanding of the mutual benefits that microbes and hosts can retrieve from such associations.

## Supplementary information

Supplementary material

## Data Availability

The Mathematica files to produce the figures are available at https://github.com/flobansept/microbes_life_history_selection.
