# Supplementary material for: Modeling host-associating microbes under selection
Source: ISME J. 2021 Jun 22;15(12):3648–56. doi: 10.1038/s41396-021-01039-0 (PMC8630024; doi:10.1038/s41396-021-01039-0)
Supplement: Supplementary file 1 — Supplementary material [file 41396_2021_1039_MOESM1_ESM.pdf]

Supplementary material

S1 Asymmetric migration rates

Let us now consider an asymmetry between the migration rates. Setting $m_E = m$ and $m_H = \alpha m_E$, the expression of the overall growth rate becomes

$$\lambda_a = \frac{1}{2}\left(1 + r_H - (1+\alpha)m + \sqrt{(m(1+\alpha) - 1 - r_H)^2 - 4(r_H - m(1+\alpha r_H))}\right).$$

The resulting shape of the fitness landscape is qualitatively unchanged: λ is still maximized for low values of the migration rates and high values of the replication rates ratio, and the maximum value for the overall growth rate λ is still 1 (in units of r_E). However, the depth of the landscape changes with α: the larger α (*i.e.* the more migration towards the host is favored compared to migration into the environment), the larger the amplitude of the landscape, and the steeper the slope to climb to reach the maxima. How does this translate in terms of optimal strategy? Figure S1 shows how the contour delimiting the optimality of the two strategies is modified. When α is increased the range of optimality of strategy II (decreasing m_H) is narrowed. This is expected, because setting a large α relatively increases m_H. For $\alpha > 2$, a third optimal strategy (increasing m_E) appears between the two original ones, because the asymmetry between the initial values of the migration rates makes it more efficient to act upon m_E than m_H.

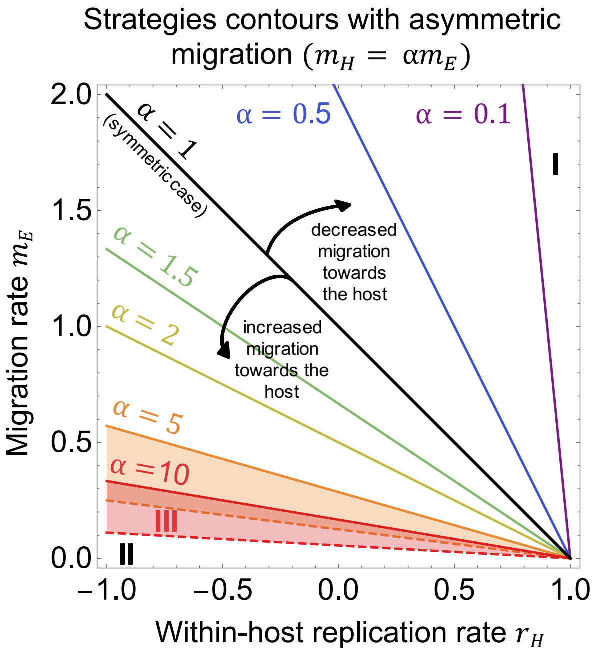

Figure S1: Change in the contour line delimiting the regions of optimality of the two strategies defined in Figure 1C with α, the ratio of the migration rates. The black thick line shows $\alpha = 1$, *i.e.* the symmetric case identical to the contour shown in Figure 1C. Increasing the migration towards the host is unfavorable for the considered lineage, because of the assumption that $r_H \leq r_E$, thus decreasing the area of optimality of strategy II. For $\alpha > 2$, a region where strategy III (increasing m_E) is optimal appears between the strategies I and II (shown by the shaded areas).

S2 Global competition: supplementary figure

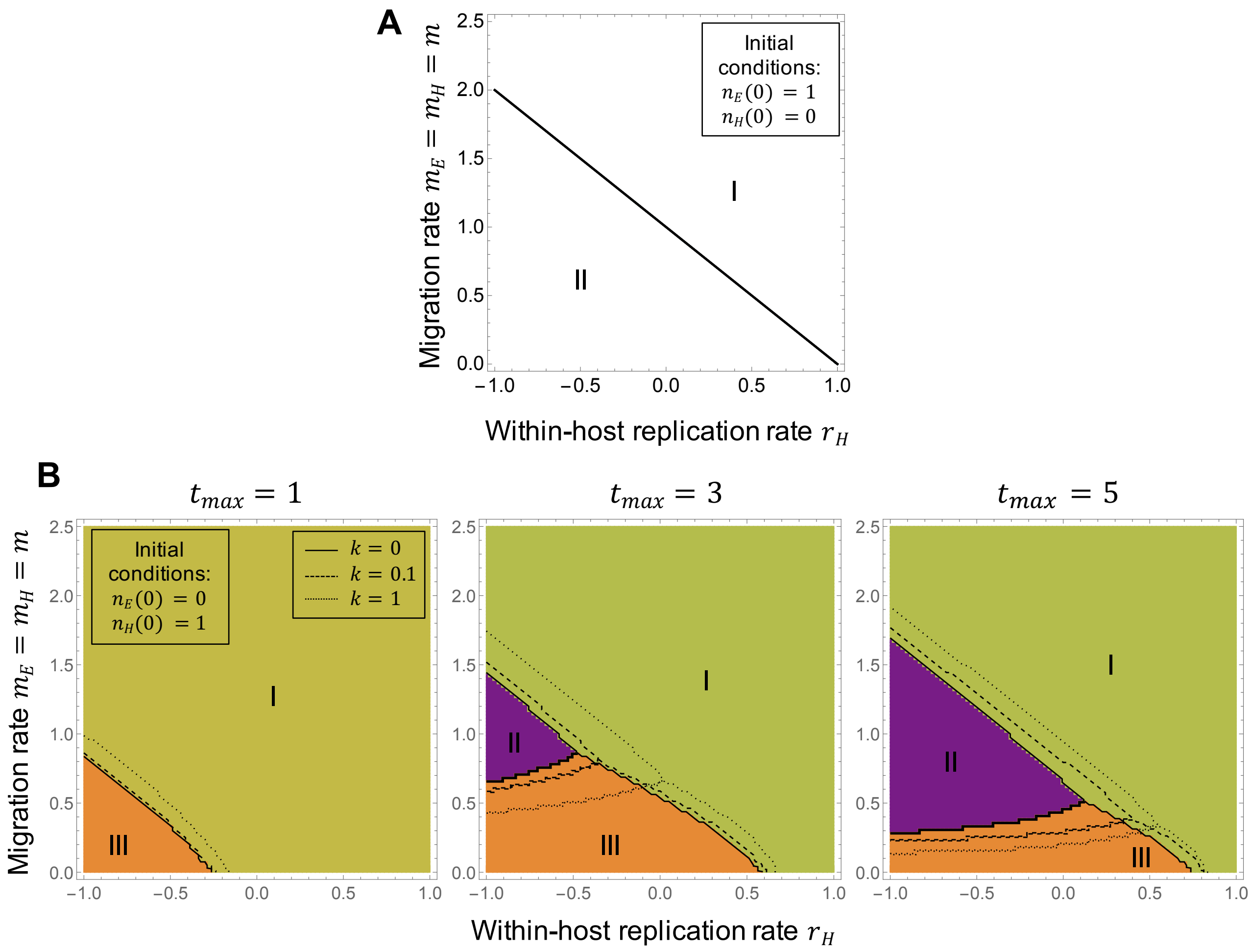

Figure S2: **Contour lines delimiting the regions of optimal strategies in the model with global competition.** The initial conditions are the two stage-biased vectors: the host is initially empty in panel (A), while the environment is initially empty in panel (B). In panel (A), all the contour lines collapse to the baseline asymptotic limit (the different t_{max} and k shown are identical to the ones in Figure 2B). In panel (B), a third optimal strategy (increasing m_E) appears.

S3 Competition in the host only: supplementary figure

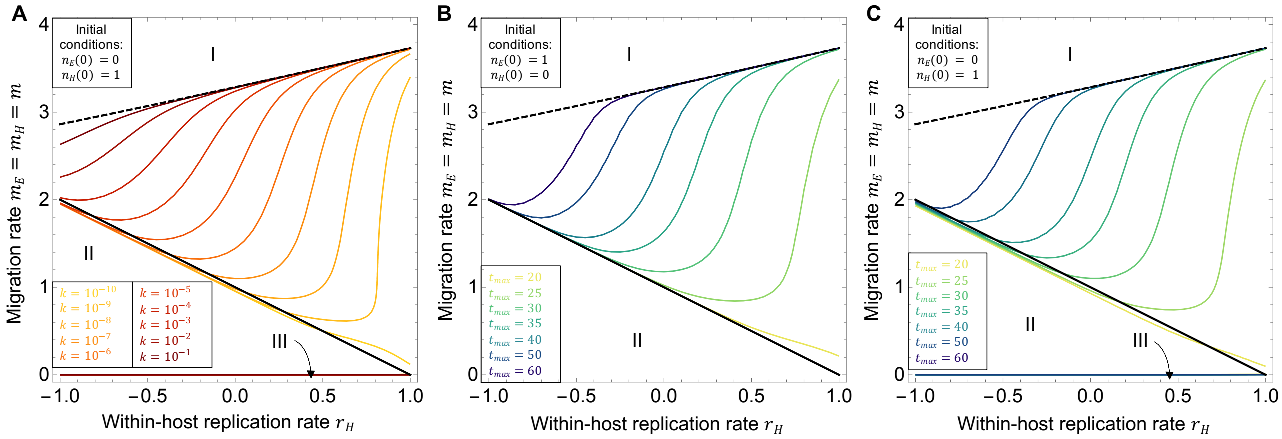

Figure S3: **Contour lines delimiting the regions of optimal strategies in the model with competition in the host only**. Changes of the contours with k (within-host competition intensity) (A) and with t_{max} (B and C). All the microbes are initially in the host ($n_E(0) = 0, n_H(0) = 1$) for panels A and C, while they are initially in the environment for panel B. Solid colored lines: limit between the regions of optimality of strategy I (increasing r_H) and II (decreasing m_H) or II and III (increasing m_E). Other parameters: $t_{max} = 30$ (A) and $k = 10^{-7}$ (B and C). With increasing k or t_{max}, the region of optimality of strategy I tends to narrow down and shifts out of the $m < 1$ region to converge to the contour of equal sensitivities of the number of microbes at equilibrium (dashed line).

S4 Competition in the environment only: supplementary figures

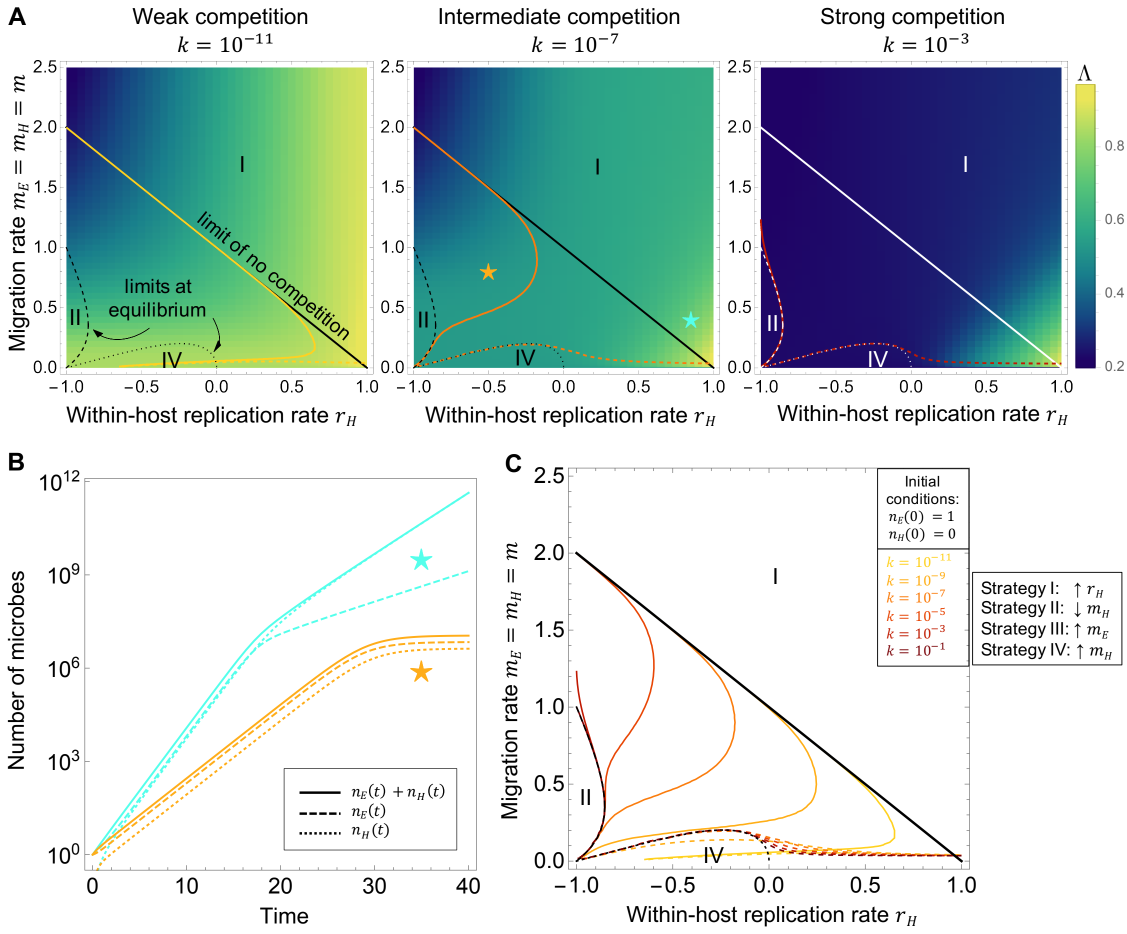

Figure S4: **Optimal strategies in the model with limited growth in the environment only**. **(A)** Change in the fitness landscape with the competition intensity $k = k_{EE}$. Colored lines: contours of equal sensitivities delimiting the optimal strategies (as shown in panel C): solid line, between strategies I and II; dashed line, between I and IV. Black lines: limit contours (solid line: limit of no competition, from the base model; dashed and dotted lines: limit derived from the number of microbes at equilibrium). Other parameters: $t_{max} = 30$, $n_E(0) = 1, n_H(0) = 0$. **(B)** Number of microbes in function of time, for the parameters combinations indicated by colored stars in panel A. When there is competition in the environment only, there is an equilibrium only if $m_E > m_H$. **(C)** Change in the contour lines delimiting the regions of optimality of the strategies with increasing k (competition intensity). All the microbes are initially in the environment ($n_E(0) = 1, n_H(0) = 0$). Solid colored lines: limit between the regions of optimality of strategy I (increasing r_H) and II (decreasing m_H); dashed colored lines: between strategy I and IV. Other parameter: $t_{max} = 30$. The region of optimality of strategy I tends to expand until it converges to the contour of equal sensitivities of the number of microbes at equilibrium (black dashed line). The black dotted line is also derived from the number of microbes at equilibrium and delimits the area of optimality of strategy IV.

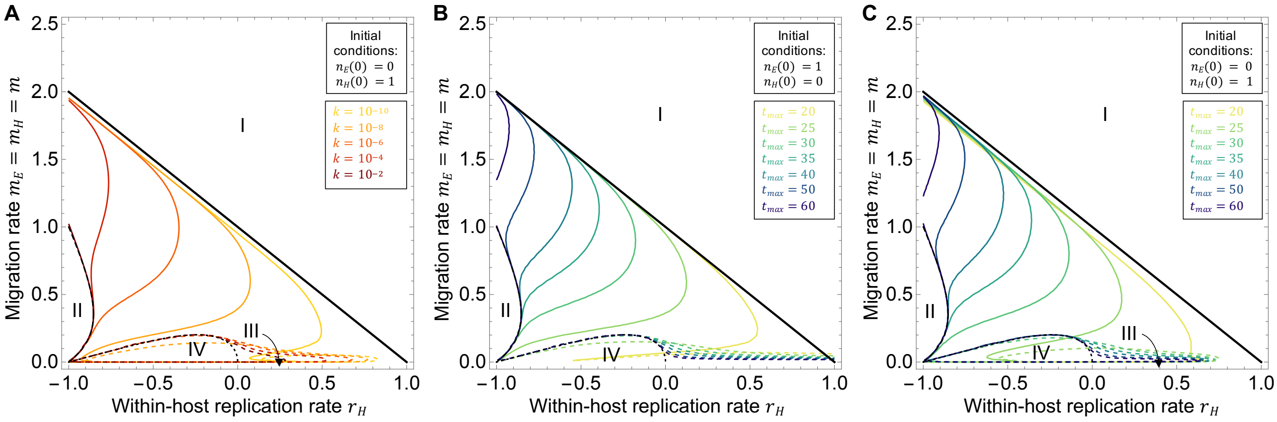

Figure S5: **Contour lines delimiting the regions of optimal strategies in the model with competition in the environment only**. Changes of the contours with k (environmental competition intensity) (A) and with t_{max} (B and C). All the microbes are initially in the host $(n_E(0) = 0, n_H(0) = 1)$ for panels A and C, while they are initially in the environment for panel B. Solid colored lines: limit between the regions of optimality of strategy I (increasing r_H) and II (decreasing m_H). Dashed colored lines: between the strategies I and IV (increasing m_H) or IV and III (increasing m_E). Other parameters: $t_{max} = 30$ (A) and $k = 10^{-7}$ (B and C). With increasing k or t_{max}, the region of optimality of strategy I tends to expand until it converges to the contour of equal sensitivities of the number of microbes at equilibrium (dashed black line).

S5 Competition of equal intensity within each compartment: supplementary figure

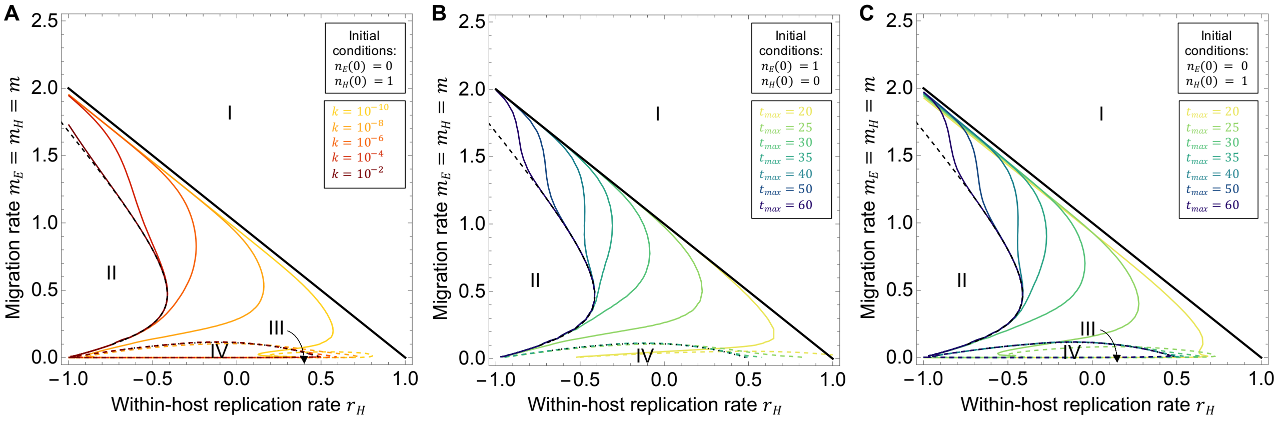

Figure S6: **Contour lines delimiting the regions of optimal strategies in the model with competition of equal intensity within each compartment**. Changes of the contours with $k = k_{EE} = k_{HH}$ (competition intensity) (A) and with t_{max} (B and C). All the microbes are initially in the host $(n_E(0) = 0, n_H(0) = 1)$ for panels A and C, while they are initially in the environment for panel B. Solid colored lines: limit between the regions of optimality of strategy I (increasing r_H) and II (decreasing m_H). Dashed colored lines: between the strategies I and IV (increasing m_H) or IV and III (increasing m_E). Other parameters: $t_{max} = 30$ (A) and $k = 10^{-7}$ (B and C). With increasing k or t_{max}, the region of optimality of strategy I tends to expand until it converges to the contour of equal sensitivities of the number of microbes at equilibrium (dashed black line).
